# Structural Genomics on the Web

**DOI:** 10.1002/cfg.79

**Published:** 2001-04

**Authors:** Jo Wixon

**Affiliations:** Bioinformatics Division HGMP-RC Hinxton, Cambridge CB10 1SA UK

## Abstract

In this review we provide a brief guide to some of the resources and databases that can be used to locate information and aid research in the growing field of structural genomics. The review will provide examples, for less experienced users, of what can be achieved using a selection of the available sites. We hope that this will encourage you to use these sites to their full potential and whet your appetite to search for other related sites.


**Comparative and Functional Genomics**
*Comp Funct Genom* 2001; **2**: 103–113.
**DOI**: 10.1002/cfg.79

**Website Review**

### Structural genomics on the web

Jo Wixon, Managing Editor
*Bioinformatics Division, HGMP-RC, Hinxton, Cambridge CB10 1SA, UK*

### Abstract

**In this review we provide a brief guide to some of the resources and databases that can be used to locate information and aid research in the growing field of structural genomics. The review will provide examples, for less experienced users, of what can be achieved using a selection of the available sites. We hope that this will encourage you to use these sites to their full potential and whet your appetite to search for other related sites. Copyright © 2001 John Wiley & Sons, Ltd.**

### Introduction

The majority of three-dimensional (3D) structures are determined using X-ray crystallography and the remainder are mainly determined using nuclear magnetic resonance (NMR). There are also computational approaches for the prediction of folds and domain structures. Homology modelling is the fitting of a known protein sequence to the experimentally determined 3D structure of a homologous protein. This method is by no means rock solid, but is usually more reliable than results derived purely from *ab initio* (theory only) modelling.

For X-ray crystallography, perfect quality, single crystals are the first requirement. This can prove immensely problematic and is widely considered to be somewhat of an art. In addition to this, the protein must be crystallised in such a way that the final lattice allows for the production of high resolution data (ideally, less than 2 Angstroms). This step has so far formed an impenetrable barrier to the determination of the structures of several important proteins. Proteins with highly hydrophobic regions cannot usually be crystallised, and this, coupled with the difficulty in purifying such proteins, is the cause of the paucity of transmembrane protein data in resources such as the Protein DataBank (PDB, see below). In recent times this problem has been overcome to some extent by cloning and expressing only those parts of genes that encode the soluble portions of such proteins.

Crystallisation can distort parts of a structure due to contacts between neighbouring molecules in the crystal, however, the protein crystals used for diffraction studies are highly hydrated, thus the structures determined from crystals are often not far removed from those of the proteins in aqueous solution. During X-irradiation of the crystal, electrons in the crystal diffract the X-rays, producing a diffraction pattern. This can be computationally processed to generate an electron density map, which is characteristic of the protein structure. Using knowledge of the primary sequence of the protein, the amino acids are fitted into the map to form a model of the structure. This model is then refined a number of times, in an iterative process, before the final structure is accepted. Regions of a chain that are mobile (or disordered) provide poor results and cannot usually be assigned; it is quite common for the N and C-terminal regions and small loops of proteins to fall into this category. The generation of the electron map from the diffraction data is a complex process; this can be aided by also determining the diffraction pattern of a crystal with a bound heavy metal ion (provided that this does not disrupt the structure). A more recent approach is the use of synchrotron radiation at multiple wavelengths, which has speeded up the process of solving structures.

A drawback of this technique is that it usually cannot resolve the positions of hydrogen atoms or distinguish between nitrogen, oxygen and carbon reliably. This means that it is not possible to identify the side-chains for some amino acids and usually, this information has to be inferred from other data. Only in those rare cases where the resolution of the

Copyright © 2001 John Wiley & Sons, Ltd.

data extends beyond ~1.2 Angstroms has it been possible to locate some of the hydrogen positions based on the X-ray diffraction data.

NMR is used to determine the structures of proteins in solution, and is dependant upon the presence of atomic nuclei with a spin. The method detects chemical shifts of these atoms as they tumble and vibrate whilst in solution. Hydrogen is one such atom that occurs naturally in proteins, but better data are obtained when others ($^{13}C$ and $^{15}N$) are used to chemically label the proteins before determining their structures. The chemical shifts are determined by the environment of the atoms, which is determined by what types of nuclei are nearby and at what distances. The result of an NMR analysis is a set of constraints; these are estimates of the distances between pairs of bonded or non-bonded neighbouring nuclei. If enough of these are obtained, then the number of possible structures that fit these constraints is finite. Commonly, these remaining models are then averaged and adjustments are made to accommodate bond lengths and angles. The result is a family, of perhaps 10–50 models. Where the models match well, the constraints have tightly defined the structure, where there is more variation, this can be due to more mobile parts of the structure, such as the N and C-terminals and loops which are more free to move around.

Restrictions on this method are that the protein must tumble rapidly, hence it is limited to molecules not much greater than 30 kD, and the protein must be highly soluble and stable (i.e. not tend to aggregate during storage). NMR is typically the method of choice for small proteins that are not readily crystallized and it does yield the positions of some hydrogen atoms. NMR in detergent can circumvent some of the problems in purifying transmembrane proteins and has thus provided structures for some of these proteins, which could not be solved by X-ray crystallography.

Whereas crystallography yields a unique model for the structure, NMR analysis produces results that are a combination of alternative models. There are cases where molecules have been studied both by crystallography and solution NMR, and the agreement has been very good indeed, resulting in a high level of confidence in these structures.

It is unclear as yet how, or indeed if, it will be possible for either of these techniques to ever really become a high throughput, systematic approach. There have been some advances in this area and a few consortia have now come together to investigate ways of speeding up the process. There are also several initiatives aimed at spreading the task of determining the majority of structures from chosen organisms. One crucial issue here is the need to choose targets wisely, and careful co-ordination of these projects allows them to avoid too much duplication of effort, by choosing one gene per predicted structural family and keeping up-to-date records of which targets are under study by which members.

In industry, the hopes for achieving high throughput structure determinations are higher. The companies involved combine access to synchrotron facilities with their experience in converting protein expression and purification strategies into high-throughput systems. Their aim is to apply these techniques to accelerate the drug discovery process.

### Resources on the web

### Databases

There are various types of database available that hold information relevant to structural genomics, storing information on domain structures predicted from sequence data, alongside actual 3D structure information determined using X-ray crystallography or NMR. Several different approaches have been applied to categorising the proteins, from sequence alignments that take account of structural features, to visual comparisons of experimentally determined structures.

The Research Collaboratory for Structural Bioinformatics (RCSB, **http://www.rcsb.org/index.html**) describes itself as 'a non-profit consortium that aims to improve understanding of the function of biological systems through the study of the 3-D structure of biological macromolecules'. The Protein DataBank (PDB, **http://www.rcsb.org/pdb**) is provided by the RCSB. This impressive resource contains structural data obtained using X-Ray crystallography (>80%), NMR (~16%) and theoretical modelling (~2%), amounting to 14510 structures, as of 27 February this year. PDB can be searched using PDB ID numbers or two search tools. The 'SearchLite' tool allows keyword searching and is ideal for first-time users, regular visitors most likely prefer the advanced search tool 'SearchFields'. Searching with a keyword, such as 'TIM barrel' yields a table of results (Figure 1). The 'EXPLORE' links lead through to the Structure Explorer tool, in which 'Summary Information' on the chosen protein is presented (Figure 2). The left

Copyright © 2001 John Wiley & Sons, Ltd. 

frame offers several other options, including 'View Structure' which links through to a page offering a selection of display options from still images of ribbons or cylinders up to interactive immersive diagrams (some of these require the viewing program Chime). The 'Structural Neighbours' option leads to a page that will automatically query a collection of structural databases (including SCOP and CATH) for your protein of interest. The 'Other Sources' link leads through to a categorised table detailing the entries for your selected protein in related databases. There are also links to geometry information and to download the file on that protein structure.

The SCOP (Structural Classification of Proteins) database (**http://scop.mrc-lmb.cam.ac.uk/scop/**) holds an array of information on all proteins whose structure is known. The proteins have been classified manually (assisted by tools), by visual inspection and comparison of structures. The proteins are classified according to both structural and evolutionary relatedness. The hierarchy used consists of three main levels; 'family', in which members have clear evolutionary relatedness, 'superfamily', in which members may have low sequence identities, but have conserved structural and functional features, and 'folds', in which members share some major secondary structures (and their arrangement and topology), but may not have common evolutionary origins.

Like SCOP, CATH (**http://www.biochem.ucl.ac.uk/bsm/cath_new/index.html**) is a hierarchical classification of protein structures, which uses PDB as its source. From there, only those crystal structures with resolution better than 3.0 angstroms and NMR structures are selected. Then the protein structures are divided into domains (where possible) based on the results of three independent algorithms. The hierarchy used to classify the domains has four major levels; Class, Architecture, Topology (fold family) and Homologous superfamily. There are three major classes; mainly-alpha, mainly-beta and alpha-beta, and a smaller class, of domains with low secondary structure content. The architecture level is assigned manually and takes into account the orientation of the secondary structures

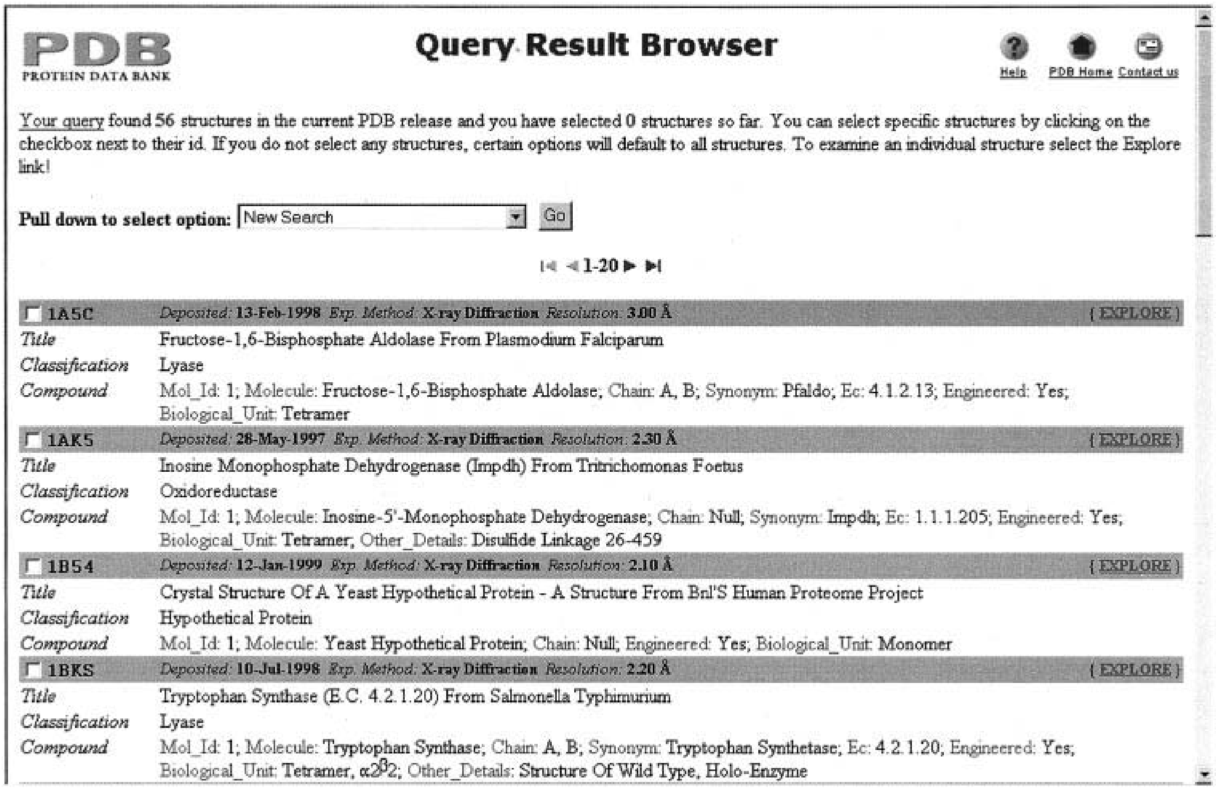

**Figure 1.** The results table for a 'SearchLite' search of PDB using the keyword 'TIM barrel'.Reproduced with kind permission of Phoebe Fagan (PDB Intellectual Property Response Section)

Copyright © 2001 John Wiley & Sons, Ltd. 

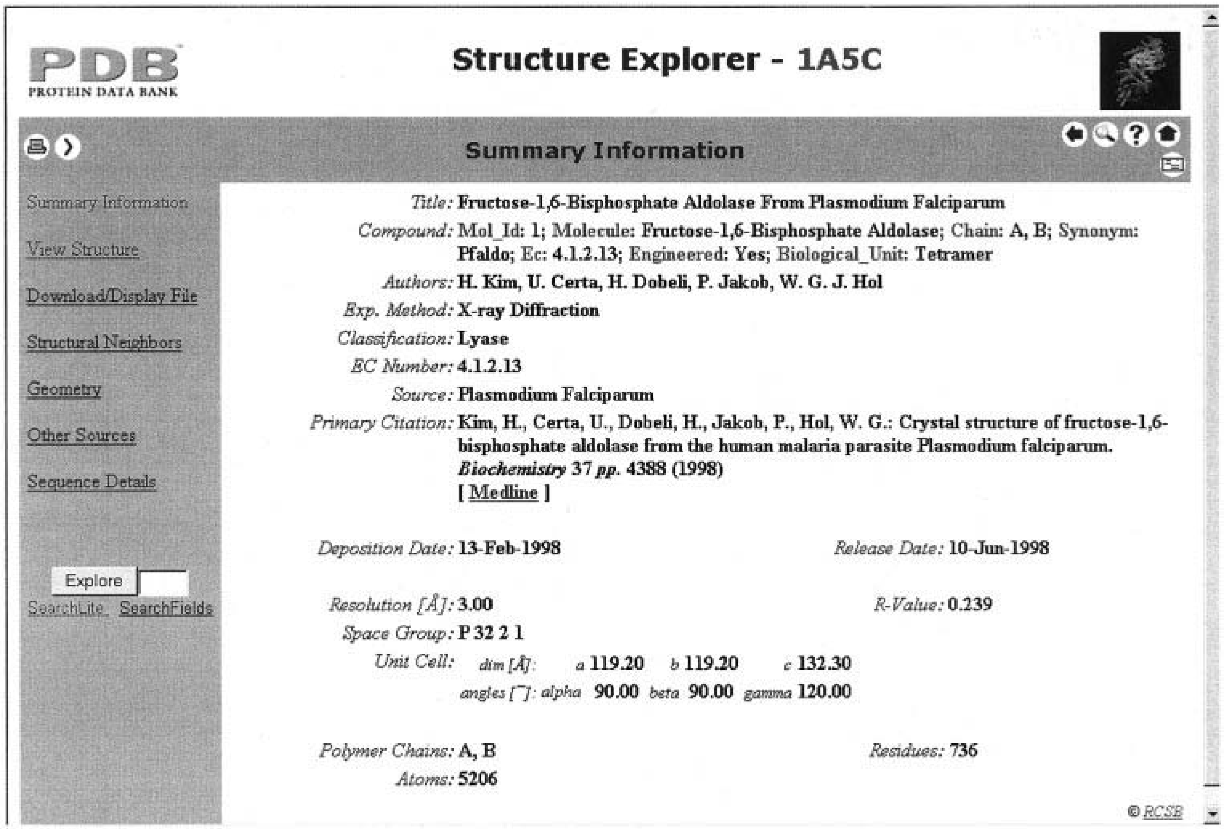

**Figure 2.** The PDB 'Structure Explorer' window for Fructose-1,6-Bisphosphate Aldolase from *Plasmodium falciparum*. Reproduced with kind permission of Phoebe Fagan (PDB Intellectual Property Response Section)

relative to one another. The topology level accounts for the shape and connectivity of the secondary structures and is determined using a structure comparison algorithm. The final level groups together domains that are believed to have a common ancestor (homologous) based upon the results of sequence and structure comparisons.

The Database of Structural Motifs in Proteins (DSMP, **http://www.cdfd.org.in/dsmp.html**) consists of domain records corresponding to ~13 000 protein entries taken from PDB. These have been categorised by structural motifs, from $\beta$-turns to barrels. If your interest is in finding all those proteins having a particular structural motif, then this seems a good place to start.

The HOMologous STRucture Alignment Database (HOMSTRAD) **http://www-cryst.bioc.cam.ac.uk/data/align/** holds structural alignments for protein families. The authors have combined classifications used by databases including SCOP, Pfam and PROSITE with the results from sequence similarity searches using PSI-BLAST and their own protein homology recognition tool, FUGUE, to make their own decisions for defining families. They aim to produce sets of protein sequences where functionally and structurally important residues are correctly aligned and then highlighted according to certain criteria. In cases where a highly conserved local sequence motif is shared by a diverse group of proteins, they may split the group into several smaller 'families'. In contrast, some families in HOMSTRAD are protein pairs with fairly low overall sequence similarity but which present convincing structure-based alignments. One way to explore HOMSTRAD is to use the 'Browse Families' option. This leads to a choice of listing the families by structural class or by family name. Choosing the 'zinc finger – CCHC-type' family results in a table (Figure 3) detailing the members of this family. The text link '1ncp' in the left hand column leads to a table of further data generated on the family members, and to their entries in other protein structure databases. Several options

Copyright © 2001 John Wiley & Sons, Ltd. 

for viewing the alignment of the chosen family are also given, one of these being 'joy-html', where a second tool from this team, called JOY, has been used to annotate the alignments according to the structural importance of the residues (Figure 4). The home page also offers the opportunity to run a quick BLAST search against the HOMSTRAD database, to run a FUGUE analysis or to use the JOY annotation tool.

The PRESAGE database (**http://presage.berkeley.edu/**) is a tool for keeping track of structural knowledge of proteins. Users create an account for themselves and afterwards can login and add or edit entries, to accumulate information on any chosen

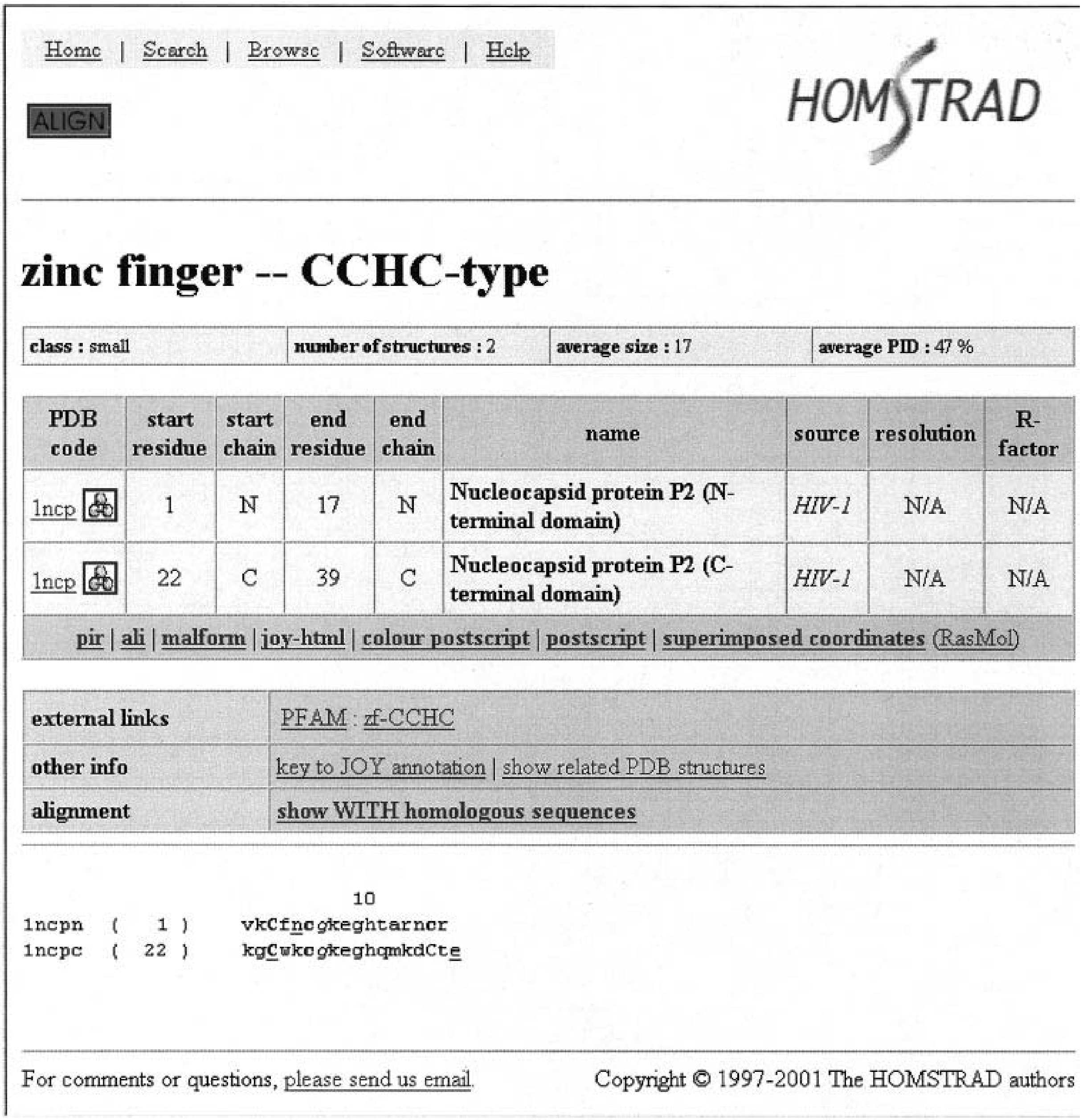

**Figure 3.** The HOMSTRAD 'zinc finger – CCHC-type' family page. Reproduced with kind permission of Dr Kenji Mizuguchi (University of Cambridge)

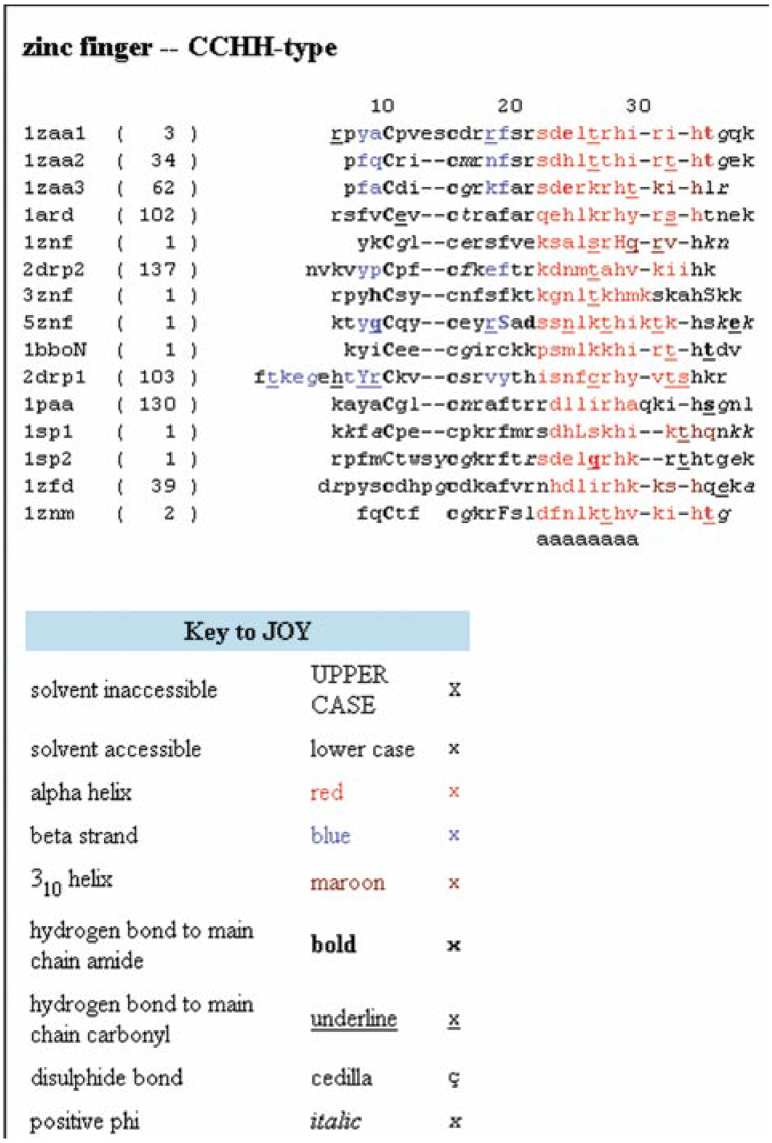

**Figure 4.** The HOMSTRAD 'joy-html' view of the 'zinc finger – CCHH-type' family alignment. The key to the annotation applied by JOY is included beneath the alignment. Reproduced with kind permission of Dr Kenji Mizuguchi (University of Cambridge)

protein. There are annotation fields for experimental data, structural predictions and models, and suggestions. The structural predictions field has a table showing the positions of any domains that have been detected in the chosen protein and has links to the relevant entries in PDB or SCOP.

BioMagResBank (**http://www.bmrb.wisc.edu**) holds NMR data on proteins, peptides and nucleic acids. The site offers five different grids to aid users in browsing the data. These include NMR spectral parameters (for chemical shift and coupling constant data), kinetics, thermodynamics and structures. The site is also home to a small suite of tools for working with NMR data and there is a good categorised list of links to other NMR web pages.

### Tools

The UCL Biomolecular Structure and Modelling group use their website (**http://www.biochem.ucl.ac.uk/bsm/index.html**) to provide public access to a wide range of tools and databases for protein sequence and structure analysis, and it is the home of the CATH database (see Databases). Some of the programs are for prediction of protein structure from sequence, others are for working with experimentally defined structures.

The Collaborative Computing Project Number 4 (CCP4), in protein crystallography (**http://www.dl.ac.uk/ccp/ccp4/**), has yielded a suite of programs that cover most of the computations required for macromolecular crystallography. The collaboration was initially a UK initiative but has since been widened to include the whole of Europe, and the site is mirrored in the US and Japan.

Dali (**http://www2.ebi.ac.uk/dali/**) is a tool for comparing protein structures in 3D. Users submit the coordinates of their query protein structure and Dali compares them with those in PDB. Once this is completed, a multiple alignment of structural neighbours is mailed back to the user. This is likely to be a valuable tool in the analysis of proteins of unknown function, since there are already several cases where comparing 3D structures has revealed biologically interesting similarities that were not detected by sequence alignments. The Dali site at the European Bioinformatics Institute (EBI) also hosts the 'Fold classification based on Structure-Structure alignment of Proteins' database (FSSP, **http://www2.ebi.ac.uk/dali/fssp/**). This is an exhaustive all-against-all 3D structure comparison of protein structures in PDB, which is automatically maintained and continuously updated using the Dali search engine. This database is very helpful for users wanting to know which proteins are the structural neighbours of a protein of interest with an entry in PDB. The EBI site is also home to the Protein Topology home page (TOPS, **http://www3.ebi.ac.uk/tops/**). This site is dedicated to topology cartoon representations of protein structures. These cartoons are used to represent secondary structure elements ($\alpha$-helices and $\beta$-sheets) and their relative orientations. An atlas, containing representative topology diagrams (generated using the TOPS program) for every protein structural group in PDB has been produced. To view a cartoon, users must choose HTML (Figure 5) or java format for viewing and then enter the PDB identifier. Users can also produce a diagram of a new protein structure by entering the structure co-ordinates that they have determined, in PDB format.

Prof. Eric Martz's page (**http://www.umass.edu/molvis/martz/**) is the home of PDB Lite and Protein Explorer. PDB Lite is a search interface for PDB, which was designed with non-specialists (who work from personal computers, unlike most structural chemists, who use Silicon Graphics workstations) in mind. Protein Explorer is a tool for exploring protein structures, again with less experienced users in mind. Protein Explorer has many useful functions, including the ability to compare two chosen structures using the 'Comparator' (Figure 6). Readers should note that this package only works with Netscape Communicator, and requires the user to download the program 'Chime', which is available from MDL Information Systems at: **http://www.mdlchime.com/chime/**. The site is also home to RasMol, a stand alone visualisation program upon which Chime (which runs inside your browser as a plug-in) was based.

### Academic projects

### Berkeley structural genomics project

**http://www-kimgrp.lbl.gov/genomics/proteinlist.html**
The Kim lab at the University of California, Berkeley is using X-ray crystallography and NMR to determine the structures of selected proteins from *Methanococcus jannaschii*, *Pyrococcus horikoshii* and *Mycoplasma pneumoniae*.

### Joint centre for structural genomics

**http://www.jcsg.org/**
This group (Stanford Synchrotron Radiation Laboratory, University of California, San Diego and The Scripps Research Institute) have chosen to study first *Caenorhabditis elegans* and then human proteins, with a focus on those involved in signal transduction, and those with novel folds. They are also investigating ways to improve the techniques used in X-ray crystallography.

### Midwest center for structural genomics

**http://www.mcsg.anl.gov/**
The main thrust of this international (USA, Canada, UK) alliance is to increase the speed of throughput in structural genomics. They are using synchrotron-based X-ray crystallography methods and aim to apply robotic technology. They are concerned with all phases of the process, from protein production and crystal growth, to structure determination and the generation and

Copyright © 2001 John Wiley & Sons, Ltd.

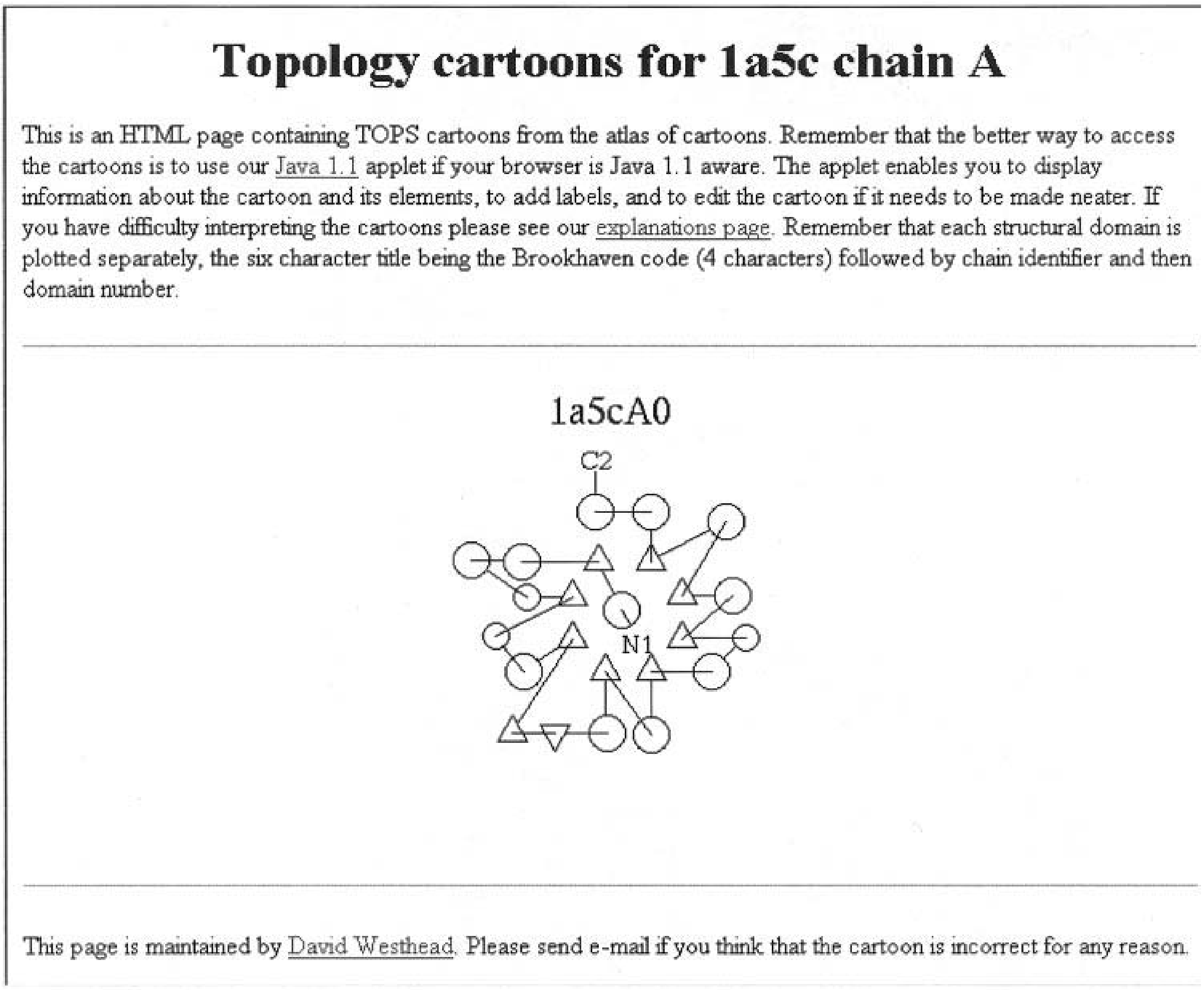

**Figure 5.** The TOPS topology cartoon for Fructose-1,6-Bisphosphate Aldolase from *Plasmodium falciparum* (PDB: 1a5c chain: A). The TIM Barrel arrangement of 8 upward oriented $\beta$-sheets (triangles) can clearly be seen, surrounded by $\alpha$-helices (circles). Reproduced with kind permission of Dr David Westhead (University of Leeds)

refinement of structural models. Their chosen targets are proteins with novel folds, or which are unique to pathogenic organisms, or unique to eukaryotes.

#### *Mycobacterium tuberculosis* structural genomics consortium

**http://www.doe-mbi.ucla.edu/TB/**

This international consortium aims to determine the structures of over 400 proteins from *M. tuberculosis*, and expects that these will include about 40 novel folds and 200 new families of protein structures. They have centralised facilities that will carry out protein production, crystallization and X-ray data collection. They aim to make the structural and functional information they obtain publicly available.

#### New York structural genomics research consortium

**http://www.nysgrc.org/**

This group are also interested in developing the technologies used in X-ray crystallography, from expression of proteins in *Escherichia coli*, through crystallisation strategies, to data collection and modelling, and further, to annotation and dissemination of data (one member of the consortium is the Brookhaven National laboratory, which is the home of PDB).

#### Northeast structural genomics consortium

**http://www.nesg.org/**

This team plan to use X-ray crystallography and NMR spectroscopy, focussing on small proteins from model eukaryotes. Their primary target organisms are

Copyright © 2001 John Wiley & Sons, Ltd. 

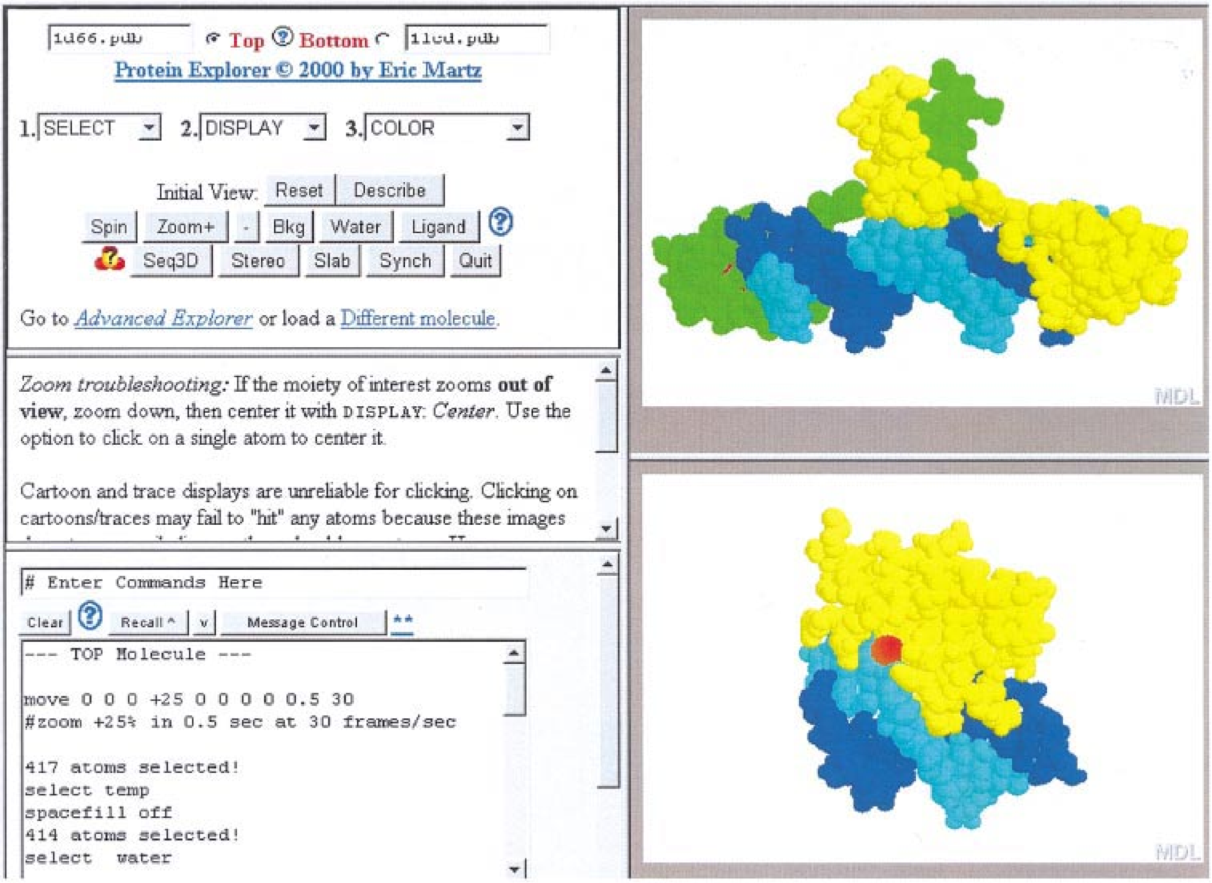

**Figure 6.** A screen capture of the 'comparator' function of Protein Explorer in action. Here, the space filling models of Gal4 (top window) and the Lac Repressor (bottom window), complexed with their DNA binding sites, are shown. The command panel allows the user to specify how the structures are presented and mouse-driven rotations or zooms of the structures can be synchronized between the two molecules. Reproduced with kind permission of Prof. Eric Martz (University of Massachusetts)

*Drosophila melanogaster*, *C. elegans*, and *Saccharomyces cerevisiae* and they plan to study some human proteins. They are also generating data for proteins from *Methanobacterium thermoautotrophicum*.

#### Protein structure initiative (PSI)

**http://www.structuralgenomics.org/**

This initiative is supported by the (NIGMS) and has contributors from an array of protein databases (COG, Pfam, PIR, ProClass, ProDom, ProtoMap, Systers and Picasso) who assign protein families and provide lists of target proteins for structural determination. There are also representatives from the Center for Advanced Research in Biotechnology and the MIT/Whitehead Institute for Genome Research. Structural genomics researchers can register with the site and create a profile in which they register their choices from the list of targets. It is also possible to check the status of a chosen protein target using its database accession number or by a FASTA search with the sequence.

#### RIKEN structural genomics

**http://www.riken.go.jp/engn/index.html**

The Protein Research Group at the RIKEN Genomic Sciences Center is studying the structure of human proteins (as domains) using NMR. They aim to compile a compendium of folds to faciliate structure prediction from sequence.

Copyright © 2001 John Wiley & Sons, Ltd.

#### Structure 2 function (S2F)

**http://s2f.carb.nist.gov/**
This collaboration between the Center for Advanced Research in Biotechnology and The Institute for Genomic Research (TIGR) is aimed at determining the structures of 50 hypothetical proteins from *Haemophilus influenzae*, to aid in determining their function. The team are using X-ray crystallography and NMR and have an annotation and modelling group.

#### The protein structure factory

**http://userpage.chemie.fu-berlin.de/ ~ psf/**
This is a German initiative that is utilising X-ray crystallography and NMR, with close links with the German Human Genome Project (DHGP). They use bio-informatic tools to help select those human proteins (or cDNAs) whose structure they will attempt to determine. The gene sequences are checked to identify those which appear to encode proteins amenable to structure determination (small, soluble, non-repetitive etc.). This is followed by structure prediction methods, e.g. 'can it be modelled upon a homologue, or does it appear to have an interesting new fold worth studying?'

#### Ontario cancer institute structural proteomics initiative

**http://nmr.oci.utoronto.ca/arrowsmith/proteomics/index.html**
This team aim to develop high-throughput methods for determining protein structures. Their initial strategy is to focus on the current rate-limiting step of the process, which is the generation of high-quality protein samples. Since modular or multidomain proteins are poor structural targets for NMR studies or to be crystallized, they have chosen to work with individual protein domains. They purify multidomain proteins and then obtain domains by limited proteolysis and trimming of termini. They are focussing on the thermophilic methanogen, Methanobacterium thermoautotrophicum.

### Industrial interests

#### Structural biology industrial platform

**http://www.sbip.org/**
This is a consortium of 15 European pharmaceutical companies, who share an interest in structural genomics. They have working groups for 3D biology, structure-function prediction, software and protein expression. The platform provides news on meetings, discussion areas and opportunities for partnering amongst its members.

#### Structural genomix

**http://www.stromix.com**
This company choose a target and then express orthologous proteins from multiple organisms, thus increasing their chances of successful crystallization, and their use of 96-well crystallisations increases throughput for that stage of the process. Their use of synchrotron radiation also improves the speed of structure determination.

#### Syrrx

**http://www.syrrx.com/**
Syrrx aim to combine their experience in high-throughput protein production and virtual ligand screening with high-throughput X-ray crystallography to accelerate the drug discovery process. Like Structural Genomix, they have guaranteed access to a synchrotron for their diffractions, which also accelerates the pace of their structure determinations.

### Conclusions

Whilst the list of web pages provided here may highlight some pages which are new to those working in structural genomics, the examples of tool and database functionality are aimed more at newcomers to the field, or those who have found out that their favourite protein is a homologue of one of known structure, or suspect that structural information may add to their understanding of its function. To view 3D structures and superimpositions of structures (as offered by the majority of the databases covered in this article) software such as 'RasMol' or 'Chime' must be loaded onto the workstation you are using, this can require a large amount of computing power, hence the widespread use of Silicon Graphics workstations amongst the community. Since many of our readers will not have this option, the examples given are mainly restricted to simpler views of the data, which will run on PCs equipped with standard Internet browsers.

Whilst some of the databases featured here are well established and highly populated, it should be noted that the field of high-throughput structural genomics is still quite young and so the majority of

Copyright © 2001 John Wiley & Sons, Ltd.

the consortium sites are as yet still under construction. There is however a huge capacity for the Internet to facilitate the work done in this field, most importantly by allowing dissemination of the data and the further development of tools for viewing structures, but not least by aiding the co-ordination of such consortia. These are exciting times for those working on protein structure determination, so keep watching these pages!

### Other useful sites/lists of links

NIGMS Structural Genomics Initiatives (and Meeting Reports)
**http://www.nigms.nih.gov/funding/psi.html**

NMR information server
**http://micro.ifas.ufl.edu/**

Structural Biology/Pharmaceutical/Protein Projects funded by 5th Framework of the EU
**http://www.sbip.org/FP5projects.htm**

### Books on protein structure

### General

Branden C, Tooze J. 1999. *Introduction to Protein Structure*. Garland Publishing.
Suelter CH. 1991. *Protein Structure Determination*. John Wiley and Sons Ltd.

### Crystallography

Clegg W. 1998. *Crystal Structure Determination*. Oxford University Press, Oxford, UK.
Glusker JP, Trueblood KN. (Eds.) 1985. *Crystal Structure Analysis*. Oxford University Press Inc., USA
Glusker JP, Lewis M, Rossi M. 1994. *Crystal Structure Analysis for Chemists and Biologists*. John Wiley and Sons Ltd. VCH.
Massa W, Gould RO. (Translator) 2000. *Crystal Structure Determination*. Springer-Verlag GmbH. Berlin and Heidelberg, Germany.
McRee DE. 1999. *Practical Protein Crystallography*. Academic Press.
Rhodes G. 1999. *Crystallography Made Crystal Clear*. Academic Press.
Rousseau JJ. 1998. *Basic Crystallography*. John Wiley and Sons Ltd.

### NMR

Cavanagh J, Fairbrother W, Palmer AG, Skelton N. 1995. *Protein NMR Spectroscopy*. Academic Press.
Evans JNS. 1995. *Biomolecular NMR Spectroscopy*. Oxford University Press, Oxford, UK.
Friebolin H. 1998. *Basic One- and Two-Dimensional NMR Spectroscopy*, 3rd Revised Edition. John Wiley and Sons Ltd.
Havel HA. 1995. *Spectroscopic Methods for Determining Protein Structure in Solution*. John Wiley and Sons Ltd.
Macomber RS. 1998. *A Complete Introduction to Modern NMR Spectroscopy*. John Wiley and Sons Ltd.
Markley JR, Opella SJ. (Eds.) 1997. *Biological NMR Spectroscopy*. Oxford University Press Inc, USA

### Acknowledgements

Source for Introduction:
Martz E. Nature of 3D Structural Data. **http://www.rcsb.org/pdb/experimental_methods.html**

---

Some of the sites reviewed will already be known to you but perhaps their content will be less well-known. The Website Review is intended to help you discover new sites of interest, but also to provide a rapid and convenient means of revealing what you always knew was there but never had the time or inclination to look at. These articles are a personal critical analysis of the Websites listed. If you have any information about sites you think are worthy of being more widely known, the Managing Editor would be pleased to hear from you.

Copyright © 2001 John Wiley & Sons, Ltd. 

